# Global resurgence and geographic expansion of SAT1 foot-and-mouth disease: insights from outbreaks in Lesotho, Cyprus and Greece

**DOI:** 10.3389/fvets.2026.1963815

**Published:** 2026-10-13

**Authors:** Raaghavi Aravindan, Paraskevi Goggolidou

**Affiliations:** Faculty of Science and Engineering, University of Wolverhampton, Wolverhampton, United Kingdom

**Keywords:** disease surveillance, Eastern Mediterranean, foot-and-mouth disease, livestock movement, outbreak epidemiology, SAT1

## Abstract

Foot-and-mouth disease (FMD) is an important transboundary animal disease affecting livestock worldwide. Analysis of outbreaks reported to the World Organisation for Animal Health (WOAH) between 2005 and 31 July 2026, with 2026 representing an incomplete year, identified 2025 as the largest outbreak year of the study period and revealed a marked increase in SAT1-associated outbreaks, whereas most other serotypes remained stable or declined. While Africa recorded its highest recent outbreak burden, Europe experienced a notable resurgence following two consecutive years without reported outbreaks (2023–2024), highlighting changing patterns in SAT1 distribution. Descriptive spatiotemporal analyses focused on three contrasting SAT1 scenarios: the first occurrence of FMD in Lesotho, the first reported SAT1 outbreak in Cyprus and the re-emergence of SAT1 in Lesvos, Greece. Reconstruction of official WOAH event reports showed that the Lesotho outbreak was repeatedly associated with illegal animal movement and epidemiologically linked cattle posts. In Europe, two distinct outbreak waves were identified, with the second characterised by SAT1 outbreaks in Cyprus and Lesvos following earlier SAT1 circulation in neighbouring Turkey. Descriptive spatiotemporal analyses demonstrated progressive geographic expansion of reported outbreaks across both islands, while comparative analyses revealed differences in species involvement and outbreak management, with vaccination predominating in Turkey and stamping-out measures used in Cyprus and Lesvos. These findings highlight the recent geographic expansion of SAT1 into previously unaffected settings and suggest that livestock movement, farm connectivity and integrated control strategies warrant further consideration for future surveillance and preparedness.

## Introduction

Foot-and-mouth disease (FMD) is a highly contagious disease affecting domestic and wild cloven-hoofed animals. Despite being recognised for centuries, it remains endemic in more than 100 countries and causes substantial economic losses worldwide. FMD is characterised by fever and vesicular lesions affecting the mouth, feet and teats, resulting in productivity losses (1). Disease severity varies among host species, with cattle exhibiting greater susceptibility and morbidity than sheep and goats (2, 3).

Seven immunologically distinct foot-and-mouth disease virus (FMDV) serotypes are recognised: O, A, C, Asia1, SAT1, SAT2, and SAT3. Serotype O remains the most widespread, whereas the SAT serotypes are historically associated with Africa (4). However, movement of animals, products and people has enabled incursions beyond their traditional range, highlighting transboundary spread into disease-free regions (5, 6).

Although Europe has remained largely free from sustained FMD transmission, sporadic incursions and outbreaks have continued. Historically, European outbreaks have been dominated by serotype O; however, renewed SAT1 activity has recently been reported in Turkey, Cyprus and Greece. Previous spatiotemporal analysis of FMD in Turkey identified spatial clustering of serotypes A, O and Asia1 and an overall northeast-to-southwest directional pattern during 2010–2019, highlighting spatial heterogeneity in the region (7). Recent regional analyses have also used spatial and spatiotemporal approaches to identify outbreak clustering and temporal changes in FMD distribution (8). Greece experienced an FMD outbreak in Lesvos in 1994 caused by serotype O, while historical WRLFMD records indicate that the previous reported SAT1 occurrence was in 1962 (9, 10, 13). In contrast, Cyprus had no previous documented SAT1 outbreaks. European control has traditionally relied on surveillance, movement restrictions, biosecurity and stamping-out, with selective vaccination (11).

The resurgence of SAT1 during 2025–2026 raises questions regarding expansion, transmission and outbreak control. Lesotho reported its first occurrence of FMD in 2026, while Europe experienced renewed SAT1 activity, including the first outbreak in Cyprus and re-emergence of SAT1 in Greece. This study aimed to characterise recent global and regional FMD and SAT1 patterns, compare SAT1 outbreaks in Lesotho, Cyprus and Lesvos and examine their spatial and temporal progression. Outbreak characteristics and control measures were also compared, with Turkey included as regional context for the European outbreaks.

## Methods

### Literature and data collection

Data were obtained primarily from the World Organisation for Animal Health (WOAH) World Animal Health Information System (WAHIS), using confirmed FMD outbreak reports available through 31 July 2026 (16). Detailed outbreak data were extracted for selected recent outbreaks in Lesotho, Germany, Hungary, Slovakia, Cyprus, Lesvos (Greece), and SAT1 outbreaks in Turkey. These countries represented contrasting epidemiological scenarios, including first occurrence, emergence, re-emergence, and regional spread. Relevant literature was retrieved from PubMed and Google Scholar using FMD, SAT1, epidemiology, surveillance, movement and control-related keywords.

Serotype and topotype information for selected SAT1 outbreaks was cross-checked with the World Reference Laboratory for Foot-and-Mouth Disease (WRLFMD) to complement WAHIS reports (17, 18). Where typing information differed in availability or resolution between sources, the available classification and associated uncertainty were retained. WAHIS data were manually reviewed and cleaned at the event level, consolidated by WAHIS event number, and aggregated for global, regional, temporal, serotype and descriptive cluster analyses.

### Global and regional outbreak analysis

Country and serotype information were extracted from confirmed WAHIS reports to characterise global and regional patterns. Records were retained according to their reported diagnostic and typing status; events without a definitive serotype assignment were retained as untyped, partially typed, pending or unsampled rather than assigned to a specific serotype. Annual reported outbreak frequencies described temporal trends and represent WAHIS events/outbreaks rather than incidence rates. Countries were grouped into four regions: Africa, Asia and the Middle East, Europe, and South America. Four reporting periods were compared: 2006–2010, 2011–2015, 2016–2020, and 2021–2026, with the final period including data through 31 July 2026; the periods therefore do not represent equal observation windows. Regional and serotype-specific frequencies were quantified to assess temporal changes in disease distribution. SAT1 outbreaks were compared by country to describe geographic distribution and reported outbreak frequency. European annual outbreak frequencies were calculated to contextualise the 2025–2026 resurgence.

### Event-level data extraction

Event-level WAHIS data were manually reviewed to extract outbreak date, location, administrative area, species affected, reported cases, susceptible animals, deaths, slaughtered/disposed animals, vaccination status, epidemiological links, and control measures. Geographic coordinates were retained where available. For Lesvos, 142 notifications were consolidated into ten descriptive geographic groupings (Mantamados, Pelopi, Napi, Ypsilometopo, Mesotopo, Kalloni, Parakoila, Mistegna, Mytilene and Anemotia) according to the specific outbreak localities reported in WAHIS. For Cyprus, 121 notifications were manually assigned to seven predefined geographic groupings (Larnaca East, Larnaca Central, Larnaca West, Nicosia South, Nicosia Central, Nicosia North and Limassol) according to their reported outbreak locations, with latitude and longitude reviewed where available. These were descriptive geographic groupings rather than formal statistical clusters; temporal information was used descriptively to describe outbreak progression and was not used as a formal criterion for group membership. Records sharing a WAHIS event number were consolidated as one event, and duplicate follow-up notifications were removed.

Definitions for outbreak-related terms, including “reported cases,” “susceptible animals,” “deaths,” “slaughtered/disposed animals,” and “vaccinated animals,” are provided in Supplementary Table S1.

### Spatial and temporal mapping

Spatial and temporal mapping was undertaken at multiple levels. SAT1 outbreaks in Lesotho were mapped using individual outbreak locations and coordinates reported in WAHIS. European outbreaks during 2025–2026 (until 31 July) were similarly mapped using individual reported locations to examine geographic and temporal distribution. For descriptive comparison, Wave 1 comprised the contiguous sequence of serotype O outbreaks reported in Germany, Hungary and Slovakia between January and March 2025, while Wave 2 comprised SAT1 outbreaks reported in Cyprus and Lesvos, Greece, between February and March 2026. These wave designations represent descriptive temporal and geographic groupings rather than formal statistical clusters. SAT1-associated outbreaks in Turkey were mapped to provide regional context for the subsequent outbreaks in Cyprus and Lesvos.

For Lesvos, individual WAHIS event locations and associated coordinates were used to reconstruct the spatial progression of reported outbreaks and assign events to the defined geographic clusters. Events reported only generally as “Lesvos” with non-specific coordinates were excluded from cluster-level mapping and corresponding event counts. For Cyprus, reported event locations and coordinates were manually reviewed and verified using Google Maps to assign events to the seven defined geographic clusters. Google Maps was used only for location verification.

### Comparative epidemiological analysis

Comparative analyses were performed using the extracted outbreak data. Reported cases, susceptible animals, natural deaths, slaughtered/disposed animals, and vaccination counts were compared across selected SAT1 outbreaks. For species-specific comparisons, reported case counts were compared across Cyprus and Lesvos clusters and Turkish events. For Lesvos, only cases from events assignable to the defined geographic clusters were included; events reported only as “Lesvos” or outside these clusters were excluded. Outbreak status and control measures were summarised to assess outbreak progression and management approaches across the selected regions. Comparisons were descriptive and based on reported event-level counts; absolute values were interpreted in the context of differences in geographic area, livestock population size and outbreak scale across the selected regions.

## Results

A total of 79 countries reported foot-and-mouth disease (FMD) outbreaks between 2005 and 2026. For comparative analyses, outbreaks were grouped into four geographical regions: Asia/Middle East, Africa, South America and Europe. Reported serotypes included O, A, Asia1, SAT1, SAT2 and SAT3, together with a smaller proportion of untyped, partially typed, pending confirmation or unsampled outbreaks.

Annual outbreak counts fluctuated throughout the study period (Figure 1A), with peaks in 2009 and 2017–2018 and lower activity during 2019–2024. In contrast, 2025 recorded the highest number of reported outbreaks, with 43 reports. By July 2026, 35 outbreaks had been reported, indicating continued high activity.

**Figure 1:**
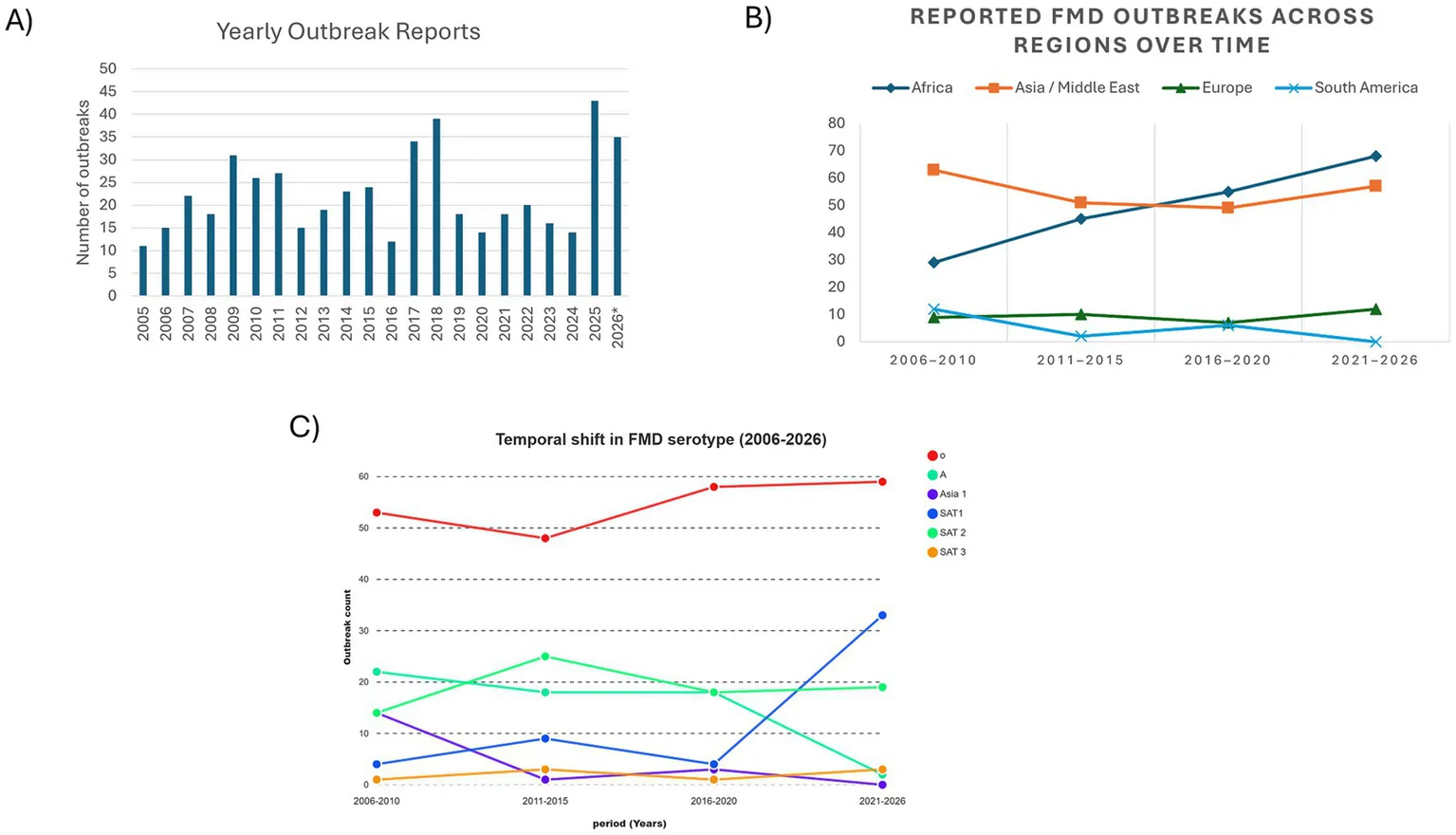
Global epidemiological trends of foot-and-mouth disease (FMD) from 2005 to July 2026. **(A)** Annual number of reported FMD outbreaks globally. **(B)** Reported outbreaks across Africa, Asia/Middle East, Europe and South America during the four reporting periods. **(C)** Distribution of reported outbreaks by FMDV serotype across the same periods. Data include WAHIS reports available through 31 July 2026; the 2021–2026 period therefore represents a partial observation window.

Regional patterns also differed substantially (Figure 1B). Africa showed a marked increase in reported outbreak burden across the study periods, rising from 29 outbreaks during 2006–2010 to 68 during 2021–2026. Asia/Middle East remained consistently high, with 63, 51, 49 and 57 reported outbreaks across the four periods, respectively. Europe remained comparatively low, although reported outbreaks increased from seven during 2016–2020 to 12 during 2021–2026. South America showed an overall decline, from 12 reported outbreaks during 2006–2010 to none during 2021–2026.

Serotype O remained the predominant reported serotype throughout the study period (Figure 1C). Across the four reporting periods, serotype O accounted for 53, 48, 58 and 59 reported outbreaks, respectively. Serotype A showed a decline from 22 outbreaks during 2006–2010 to four during 2021–2026. SAT1 showed the most pronounced recent increase, rising from four reported outbreaks during 2006–2010 to 33 during 2021–2026. SAT2 remained relatively low across the periods, while Asia1 and SAT3 also occurred at comparatively low frequencies.

Alongside the recent increase in FMD activity across Africa, SAT1 also showed spread into previously unaffected areas. Lesotho represented a notable example, reporting its first occurrence of FMD in the country in 2026, followed by first occurrences in several zones or compartments. The earliest event began in Qacha’s Nek on 5 February 2026, where suspected cases at Letlapeng were subsequently confirmed as FMDV SAT1. Illegal movement of animals was listed as the source or origin of infection and Pirbright genotyping identified topotype III (17). Further spread was recorded during March. In Leribe, beginning on 1 March, both introduction of new live animals and illegal animal movement were reported. Cases at Vuka-Mosotho, Maliba-Matso were epidemiologically linked to Matsoku through contact between cattle sharing the same mountain cattle post. In Berea, beginning on 5 March, the source was considered unknown or inconclusive, although the cases were epidemiologically linked to Leribe. The outbreak subsequently extended to Thaba-Tseka on 15 March, where illegal animal movement was again reported. In April, a direct movement associated link was documented in Mohale’s Hoek, where affected cattle had been illegally moved from Qacha’s Nek during the ongoing outbreak. Separately there is one more in same time in Butha-Buthe with no link provided (Figure 2). Overall, reported progression was associated with illegal animal movement and epidemiological links between affected areas.

**Figure 2:**
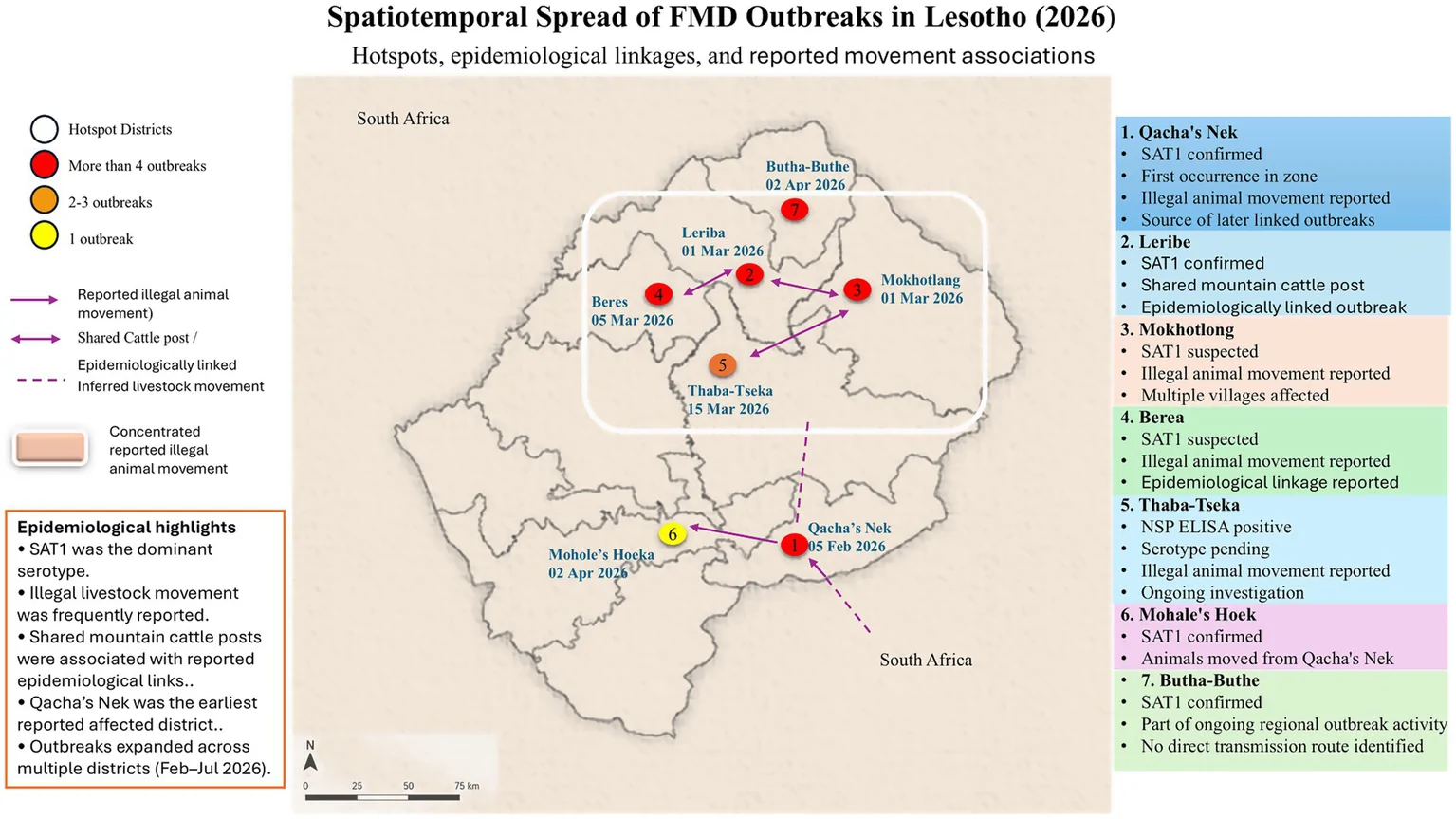
Spatiotemporal progression of foot-and-mouth disease outbreaks in Lesotho during 2026. Geographic distribution and chronology of FMD outbreaks reported across affected districts between February and July 2026 following the first occurrence in the country. Circles indicate outbreak locations, with colours representing outbreak frequency. Arrows illustrate reported illegal animal movements, epidemiological links associated with livestock movement or shared mountain cattle posts, and spatial relationships between affected districts. Boxes summarise outbreak dates, serotype status and relevant epidemiological observations for each affected district. Locations and outbreak information were obtained from WAHIS.

While SAT1 activity continued to expand within Africa, Europe also experienced a distinct FMD resurgence during 2025–2026, including an Eastern Mediterranean SAT1 wave involving areas with little or no recent history of the serotype. Similar to the global trend, a smaller outbreak wave occurred between 2011 and 2014, while the highest earlier outbreak burden was recorded in 2007, with four European countries reporting FMD outbreaks (Figure 3A). No outbreaks were reported in 2023 or 2024. This was followed by renewed activity in 2025–2026, with three European countries reporting outbreaks in 2025 and two in 2026.

**Figure 3:**
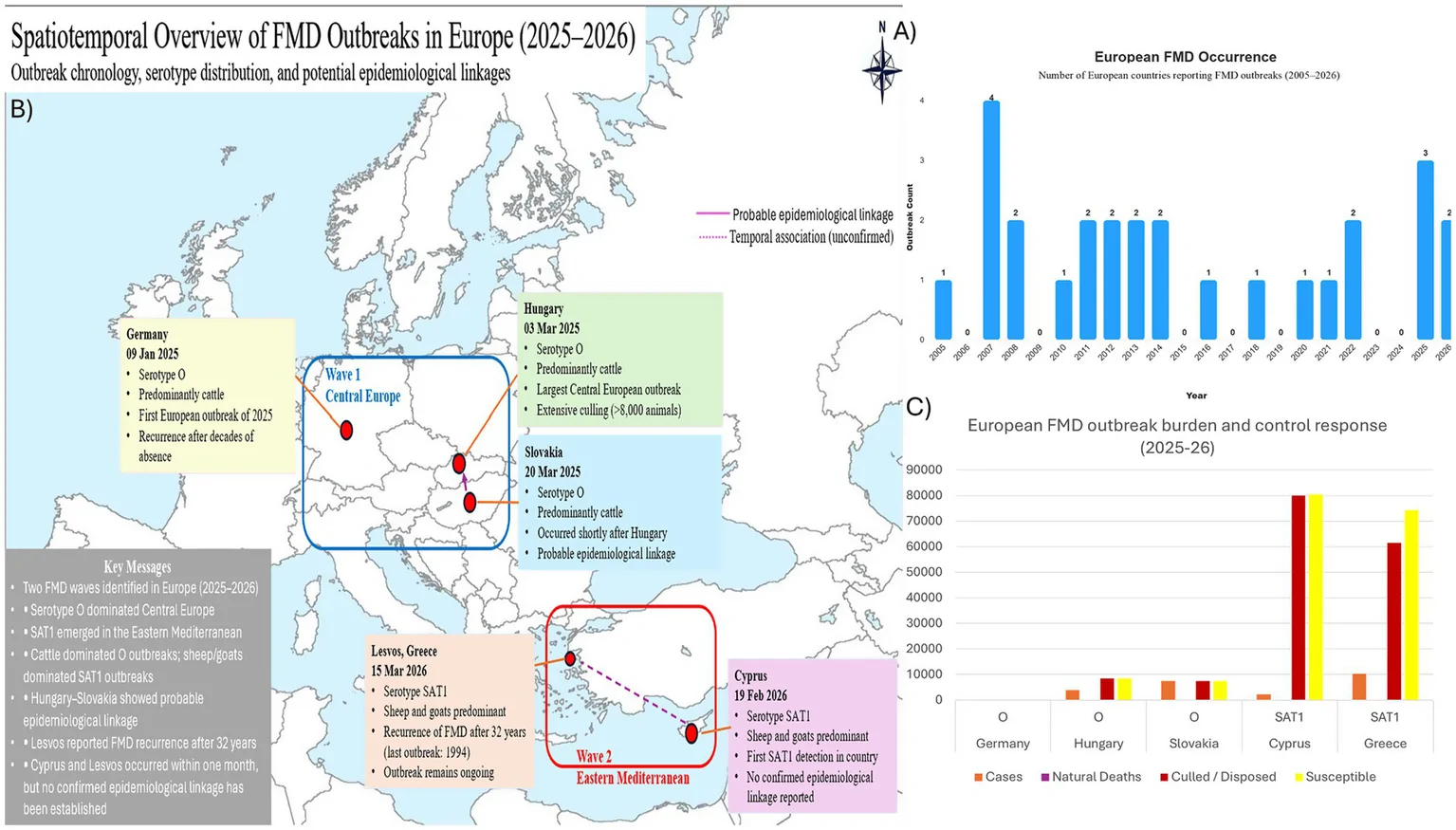
Recent foot-and-mouth disease (FMD) activity and outbreak characteristics in Europe (2025–2026): **(A)** Annual occurrence of reported FMD outbreaks in European countries between 2005 and 2026. **(B)** Geographic distribution, outbreak chronology, serotype composition, affected livestock species, and potential epidemiological relationships among reported European outbreaks during 2025–2026. Two outbreak waves were identified: a Central European wave dominated by serotype O (Germany, Hungary, and Slovakia) and an Eastern Mediterranean wave associated with SAT1 (Cyprus and Lesvos, Greece). Solid lines indicate probable epidemiological linkages, whereas dashed lines indicate temporal associations without confirmed transmission evidence. **(C)** Comparative outbreak burden and control response among affected countries during the 2025–2026 outbreak period, including reported cases, susceptible animal populations, and animals culled or disposed of as part of outbreak control measures.

The reported European outbreaks during 2025–2026 showed two descriptive temporal and geographic patterns (Figure 3B). The first occurred in Central Europe and involved serotype O outbreaks in Germany, Hungary and Slovakia. Germany reported the first event on 9 January 2025, predominantly affecting cattle and representing the first reported outbreak in the country after decades of absence. Hungary reported the second event on 3 March 2025 and recorded the largest outbreak within the Central European wave. Slovakia reported an event on 20 March 2025, also predominantly affecting cattle, with a probable epidemiological association with the Hungarian outbreak. The second pattern occurred in the Eastern Mediterranean and was characterised by SAT1-associated outbreaks. Cyprus reported the first event on 19 February 2026, predominantly affecting sheep and goats and representing the first reported SAT1 detection in the country. A second SAT1 outbreak was reported in Lesvos, Greece, on 15 March 2026, also predominantly affecting sheep and goats and representing the re-emergence of FMD after approximately 32 years. The outbreaks occurred within one month, but no confirmed epidemiological linkage was established.

Marked differences in outbreak burden and control response were observed between the two waves (Figure 3C). The Central European serotype O outbreaks involved comparatively smaller susceptible populations and culling requirements, with Hungary and Slovakia each involving approximately 8,000 animals. In contrast, the Eastern Mediterranean SAT1 outbreaks were substantially larger. Cyprus involved approximately 80,000 susceptible animals, with a similar number culled or disposed of, while Greece involved approximately 74,000 susceptible animals and more than 60,000 animals culled or disposed of. Greece recorded the highest number of reported cases among the five European outbreaks. Natural deaths remained minimal or absent across the reported events.

Although SAT1 had not been reported in Greece for several decades, SAT1 activity had previously been identified in neighbouring Turkey. Three outbreak clusters were reported in Hakkari–Van, Amasya–Çorum and Bilecik Provinces. The most recent event occurred in Bilecik Province, geographically closer to the subsequent Eastern Mediterranean island outbreaks. Although serotype confirmation was not available in the WAHIS report, available WRLFMD data identified SAT1/III in samples from the Bilecik outbreak (18). The remaining Turkish outbreaks were confirmed as SAT1, providing evidence of regional SAT1 circulation before the emergence of the Cyprus and Lesvos outbreaks (Supplementary Figure S1).

The two Eastern Mediterranean SAT1 outbreaks were examined in greater detail to compare their spatial progression, species involvement and control responses (Figure 4). In Lesvos, Greece, the outbreak began near Mantamados on 15 March 2026 and subsequently expanded across the island (Figure 4A). Early spread involved Pelopi and Napi during March, followed by Ypsilometopo in early April and westward progression through Mesotopo, Kalloni and Parakoila. Further outbreaks during May extended toward Anemotia in the west and Mistegna and Mytilene in the east. The spatial pattern indicated progressive expansion from the initial northern focus toward central, western and eastern Lesvos, with Kalloni showing a concentration of reported outbreaks. By the latest follow-up, 10,212 cases and 74,238 susceptible animals had been reported, with 61,413 animals culled or disposed of.

**Figure 4:**
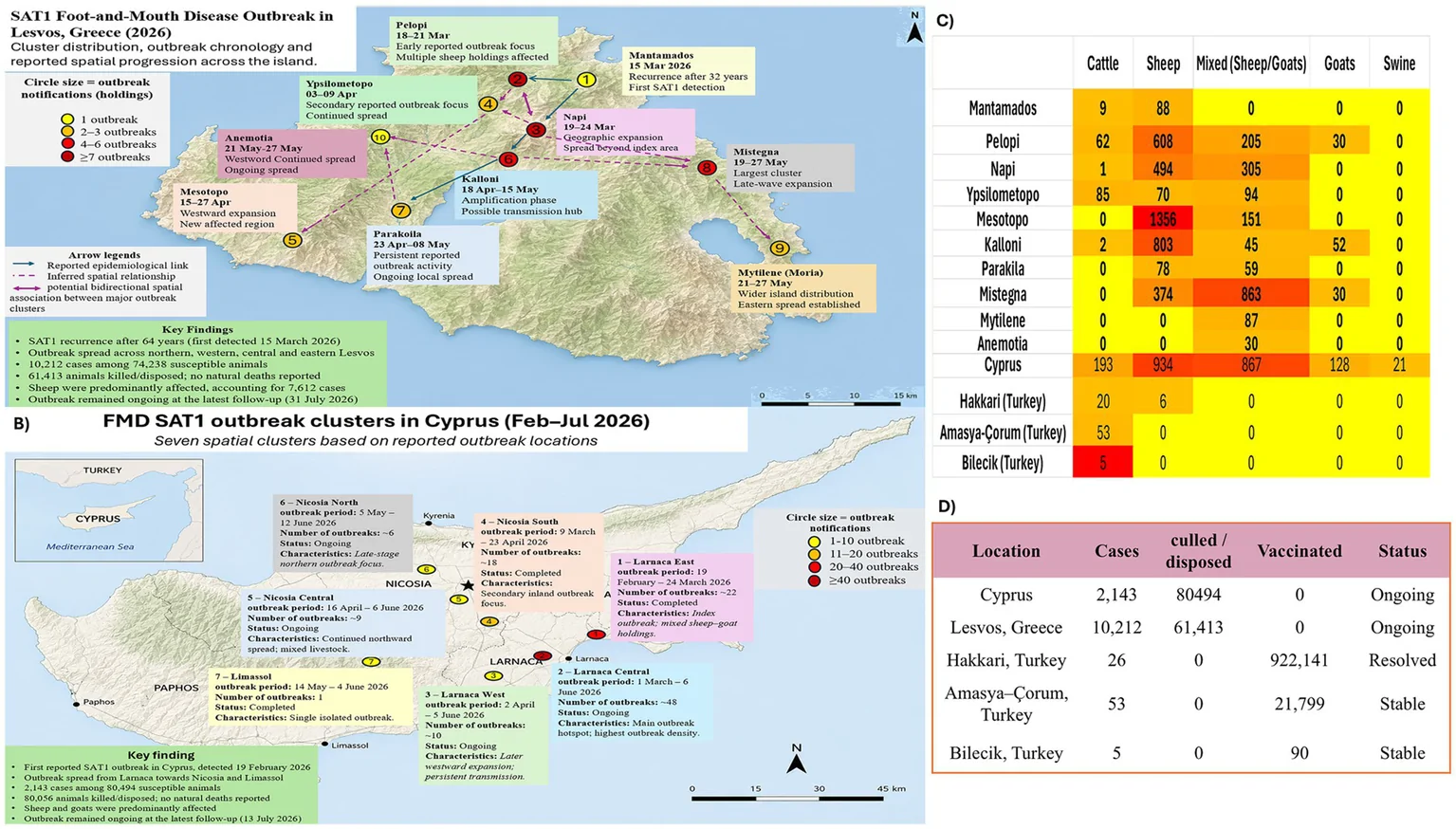
Spatiotemporal progression and comparative epidemiology of SAT1 foot-and-mouth disease outbreaks in Lesvos, Cyprus and Turkey during 2025–2026. **(A)** Spatial and temporal progression of the SAT1 outbreak in Lesvos, Greece, showing numbered outbreak clusters, chronology, and reported epidemiological or livestock movement links. **(B)** Spatial distribution and progression of the Cyprus SAT1 outbreak across seven geographic clusters from February to July 2026. **(C)** Heatmap of reported species-specific case counts across Lesvos clusters, Cyprus, and selected Turkish SAT1-affected regions. Values represent reported cases and are not standardised to the underlying livestock population or geographic area. For Lesvos, only cases from events that could be assigned to the defined geographic clusters are included; events reported only as “Lesvos” or outside these clusters were excluded. Mixed sheep–goat holdings are shown as a separate livestock category. Turkish outbreak classifications were based primarily on WAHIS, with available WRLFMD typing identifying SAT1/III in Bilecik. **(D)** Comparison of reported outbreak burden and control measures, including cases, animals culled or disposed of, vaccination, and outbreak status across Cyprus, Lesvos, and Turkey. Lesvos and Cyprus clusters are descriptive geographic groupings based on reported locations and chronology and are not formal statistical clusters. Data were obtained primarily from WAHIS, with WRLFMD used to supplement serotype and topotype information where available. Data include reports available through 31 July 2026.

A similarly extensive spatial pattern was observed in Cyprus, where the first SAT1 outbreak was detected on 19 February 2026 (Figure 4B). Seven spatial clusters were identified from reported outbreak locations. Initial activity was concentrated in Larnaca East, followed by a major outbreak focus in Larnaca Central and subsequent westward expansion through Larnaca West. Further spread involved Nicosia South, Central and North, while a single outbreak was reported in Limassol. By July 2026, Cyprus had reported 2,143 cases among approximately 80,000 susceptible animals, with 80,494 animals culled or disposed of. The outbreak remained ongoing at the latest follow-up.

The distribution of reported cases across livestock categories differed between the two island outbreaks and the preceding Turkish events (Figure 4C). The Turkish outbreaks were predominantly cattle-associated, with cattle accounting for all reported cases in Amasya–Çorum and Bilecik and the majority of cases in Hakkari. In Lesvos, cattle cases were observed primarily during the earlier outbreaks and were distributed mainly across the northern and central parts of the island, whereas sheep and mixed sheep–goat cases predominated across several subsequent clusters. Sheep involvement was particularly high in Mesotopo, Kalloni and Pelopi, while mixed sheep–goat cases were prominent in Napi and Mistegna. Cyprus showed a broader distribution of reported cases across livestock species, with sheep representing the largest reported livestock category, followed by mixed sheep–goat holdings, cattle and goats.

Marked differences were also observed in outbreak burden and control response (Figure 4D). The Turkish events were considerably smaller, with twenty six reported cases in Hakkari, fifty three in Amasya–Çorum and five in Bilecik, and were managed with vaccination without reported large-scale culling. Hakkari was subsequently resolved, while Amasya–Çorum and Bilecik were reported as stable. In contrast, Cyprus and Lesvos recorded 2,143 and 10,212 cases, respectively, and extensive culling or disposal of affected and susceptible animals, without vaccination. Both island outbreaks remained ongoing at the latest follow-up.

## Discussion

SAT1-associated outbreaks increased sharply during 2025–2026. Earlier peaks were dominated by serotype O, whereas the recent increase was strongly associated with SAT1, while other major serotypes remained stable or declined this increase coincided with geographic expansion. Africa recorded its highest outbreak burden during the study period, including outbreaks in previously unaffected areas, while Europe experienced two distinct outbreak waves, with SAT1 emerging in the Eastern Mediterranean. The pattern therefore involves increased SAT1 activity and expansion into new settings.

Although Africa recorded the highest outbreak burden, Lesotho presented an unusual situation. SAT1 is historically endemic throughout much of sub-Saharan Africa; however, the 2026 outbreaks included the first occurrence of FMD in the country, followed by first occurrences in several previously unaffected zones or compartments. This represents geographic expansion within an endemic continent, rather than continued local circulation. Official investigations identified illegal animal movement as a likely source of infection and several outbreaks were linked through newly introduced livestock or contact between cattle sharing mountain cattle posts. Livestock movement may therefore have contributed to the spread, although available data cannot distinguish between changes in livestock movement, improved surveillance or wider regional SAT1 circulation.

Europe presented a different epidemiological picture. A serotype O wave involving Germany, Hungary and Slovakia was followed by a second wave in the Eastern Mediterranean involving SAT1 outbreaks in Cyprus and Lesvos (14). Given the historically limited circulation of SAT1 within Europe, the origin of these outbreaks is interesting. SAT1 had already been reported in Turkey several months before both island outbreaks, providing important regional context. However, the Turkish outbreaks had stabilised before the emergence in Cyprus and Lesvos and were geographically separated from both islands. Without viral genomic evidence, there is currently no basis to establish a direct epidemiological link. The geographic position of Cyprus and Lesvos to the southwest of Turkey is noteworthy, in light of a previous spatiotemporal analysis that identified an overall northeast-to-southwest directional pattern of FMD outbreaks in Turkey during 2010–2019 (7). Although this spatial pattern provides relevant regional context, it cannot establish a transmission pathway to either island, particularly in the absence of livestock movement and viral genomic data.

The second European wave began with the emergence of SAT1 in Cyprus during February 2026, representing the first reported occurrence of the serotype in the country. This differed from Lesotho, where expansion occurred within the traditional SAT1 endemic region. In Cyprus, SAT1 appeared in an island setting with no previous reported circulation. The reconstructed clusters showed expansion from Larnaca toward central parts of the island, with most outbreaks remaining within the Larnaca–Nicosia corridor. The reports provide little information to explain this. Livestock movement, farm connectivity and viral genomic data would be particularly useful in understanding how SAT1 entered Cyprus and why its subsequent distribution followed this pattern.

The second event of the Eastern Mediterranean wave was reported in Lesvos approximately one month after Cyprus. Like Cyprus, Lesvos is an island separated from mainland Europe, with Turkey as a neighbouring country where SAT1 had been reported several months earlier. The history, however, was different. Greece previously experienced an FMD outbreak on Lesvos in 1994 caused by serotype O, while historical WRLFMD records indicate that the previous reported SAT1 occurrence in Greece was in 1962. Detailed WOAH notifications allowed reconstruction across multiple clusters. Whether the 2026 SAT1 outbreak represents a new introduction or re-emergence associated with wider regional circulation is unclear. The outbreak demonstrates that FMD can re-emerge on Lesvos after decades.

The pattern within Lesvos raises a question: why did the outbreak expand through the central, western and eastern parts of the island, while relatively little activity was reported in the south? Several clusters, particularly Pelopi, Kalloni and Mistegna, accounted for a substantial proportion of reported outbreaks and were distributed across different parts of the island. Claims of illegal animal movement and movement between epidemiologically connected holdings were reported but have since been disputed. This could suggest that livestock movement and farm connectivity may have contributed to the observed pattern, although livestock density, farming practices, transport networks and local topography may also have influenced outbreak development. However, these factors could not be examined, as no recorded data currently exists.

The recent occurrence of SAT1 contrasts with the historical serotype pattern documented in Turkey. Previous spatiotemporal analysis of FMD outbreaks during 2010–2019 found that serotypes O, A and Asia1 predominated in the country (7). Together with the recent SAT1 outbreaks observed in Turkey, Cyprus and Lesvos, this contrasting pattern may indicate a changing regional serotype landscape. Whether this represents an emerging regional pattern or a series of independent introductions remains uncertain. Serotype and topotype information were cross-checked between WAHIS and available WRLFMD records (16, 17, 18). WAHIS provided the primary event-level epidemiological data, while WRLFMD provided additional typing context. WRLFMD records identified SAT1 topotype III for Lesotho and in samples from the Bilecik outbreak in Turkey (16, 17, 18), while SAT1 topotype III was consistently reported for Cyprus. Available reporting also identifies the Lesvos outbreak as SAT1/III (WOAH). Broader WRLFMD surveillance indicates additional SAT1 topotypic diversity, including SAT1/II and SAT1/III among recent southern African samples, but no SAT1/I evidence was identified for the selected outbreaks examined here. In the absence of viral genomic data, these findings cannot establish a direct molecular link between the recent SAT1 outbreaks.

Species involvement also differed considerably across the Eastern Mediterranean outbreaks. The Turkish events were predominantly cattle-associated, whereas Cyprus and Lesvos were dominated by small ruminants, with Cyprus involving the broadest range of livestock species. Within Lesvos, cattle cases were observed mainly during the earlier outbreaks and were concentrated in the northern and central parts of the island, while sheep and mixed sheep–goat cases became more prominent across subsequent clusters. This could reflect differences in livestock composition and farming systems. Detection may also have contributed, as clinical signs of FMD are generally more apparent in cattle than in small ruminants. It should also be noted that both NSP ELISA and rRT-PCR were used in Lesvos, representing different types of diagnostic evidence. Differences between Cyprus and Lesvos may therefore reflect livestock distribution, surveillance and management rather than host susceptibility alone. Livestock census and movement data would help distinguish between these explanations.

Outbreak management also differed between the affected regions. Turkey incorporated extensive vaccination, whereas Cyprus and Lesvos primarily used movement restrictions and stamping-out measures without reported emergency vaccination. These approaches cannot be directly compared because outbreaks differed in geography, population, scale and epidemiological context. This study cannot determine whether emergency vaccination would have changed the course of either island outbreak. However, rapid testing and results turnaround, targeted isolation of herds and, where appropriate, targeted vaccination could potentially contribute to more rapid containment. Emergency vaccination has also been used during previous European FMD outbreaks and its potential role during rapidly expanding SAT1 outbreaks warrants further evaluation (12, 15).

Several limitations should be considered when interpreting these findings. The analysis relied on officially reported WOAH notifications, with the level of epidemiological detail varying between countries. Differences in surveillance intensity, reporting practices, diagnostic capacity, livestock population size, geographic extent and reporting scale may have contributed to variation in reported outbreak frequencies and limited direct comparison of absolute outbreak counts and control measures between case studies. The reported values should therefore be interpreted as descriptive comparisons, rather than directly standardised measures of epidemiological burden. The absence of viral genomic data prevented direct assessment of relationships between outbreaks, while the lack of livestock movement records limited confirmation of possible transmission pathways. The clusters reconstructed for Lesotho, Cyprus and Lesvos were based on reported locations and chronology and should therefore be viewed as epidemiological reconstructions rather than confirmed transmission networks. Finally, observations from these selected case studies should not be generalised to SAT1 epidemiology more broadly.

Overall, this study shows a changing epidemiological pattern for SAT1, with expansion into new settings and re-emergence after prolonged periods without reported circulation. The selected case studies illustrate how local settings, livestock movements and outbreak management may influence the pattern observed after introduction. Continued surveillance, better understanding of livestock movement and molecular epidemiological investigation will be important for reducing the risk of future SAT1 incursions in both endemic and historically disease-free regions. These findings also highlight the potential value of rapid testing and, where epidemiologically appropriate, targeted emergency vaccination as additional tools for FMD containment in Europe, warranting further evaluation.

## Data Availability

Publicly available datasets were analyzed in this study. This data can be found at: https://wahis.woah.org/#/event-management.
