# Supplementary material for: Global resurgence and geographic expansion of SAT1 foot-and-mouth disease: insights from outbreaks in Lesotho, Cyprus and Greece
Source: Front Vet Sci. 2026 Oct 13;13:1963815. doi: 10.3389/fvets.2026.1963815 (PMC13711050; doi:10.3389/fvets.2026.1963815)
Supplement: Supplementary file 1 [file Table_1.DOCX]

**Supplementary Table S1. Definitions of WAHIS reporting, epidemiological and diagnostic terminology used in this study**

| **Term/category** | **Definition/use in this study** |
| --- | --- |
| Notification/report | Official disease information submitted through WAHIS. |
| Event | Individual outbreak record reported in WAHIS. |
| Outbreak | FMD occurrence represented by the corresponding WAHIS event record(s). |
| Cluster | Group of reported events assigned to a defined geographic area for the Cyprus and Lesvos spatial analyses. |
| Cases | Number of FMD cases reported for the corresponding event. |
| Susceptible animals | Number of animals reported as susceptible in the corresponding event. |
| Deaths | Number of animals reported to have died in the event. |
| Slaughtered/disposed animals | Animals reported as slaughtered or disposed of as part of outbreak control. |
| Vaccinated animals | Number of animals reported as vaccinated in the corresponding event. |
| Serotype | FMD virus serotype reported or identified for the corresponding outbreak/event. |
| Topotype | Genetic lineage classification within an FMD virus serotype, based on available WRLFMD typing information. |
| Confirmed | Event/record reported as confirmed in WAHIS. |
| Suspected | Event/record reported as suspected in WAHIS. |
| NSP-positive | Record reported as positive based on non-structural protein (NSP) testing. |
| Pending | Diagnostic status reported as pending in WAHIS. |
| Untyped | No specific FMD serotype was assigned in the corresponding WAHIS record. |
| Partially typed | Serotype information was available for only part of the reported event/records. |
